# Machine Learning Classifiers for Twitter Surveillance of Vaping: Comparative Machine Learning Study

**DOI:** 10.2196/17478

**Published:** 2020-08-12

**Authors:** Shyam Visweswaran, Jason B Colditz, Patrick O’Halloran, Na-Rae Han, Sanya B Taneja, Joel Welling, Kar-Hai Chu, Jaime E Sidani, Brian A Primack

**Affiliations:** 1 Department of Biomedical Informatics University of Pittsburgh Pittsburgh, PA United States; 2 Intelligent Systems Program University of Pittsburgh Pittsburgh, PA United States; 3 School of Medicine University of Pittsburgh Pittsburgh, PA United States; 4 Department of Linguistics University of Pittsburgh Pittsburgh, PA United States; 5 Pittsburgh Supercomputing Center Carnegie Mellon University Pittsburgh, PA United States; 6 College of Education and Health Professions University of Arkansas Fayetteville, AR United States

**Keywords:** vaping, social media, infodemiology, infoveillance, machine learning, deep learning

## Abstract

**Background:**

Twitter presents a valuable and relevant social media platform to study the prevalence of information and sentiment on vaping that may be useful for public health surveillance. Machine learning classifiers that identify vaping-relevant tweets and characterize sentiments in them can underpin a Twitter-based vaping surveillance system. Compared with traditional machine learning classifiers that are reliant on annotations that are expensive to obtain, deep learning classifiers offer the advantage of requiring fewer annotated tweets by leveraging the large numbers of readily available unannotated tweets.

**Objective:**

This study aims to derive and evaluate traditional and deep learning classifiers that can identify tweets relevant to vaping, tweets of a commercial nature, and tweets with provape sentiments.

**Methods:**

We continuously collected tweets that matched vaping-related keywords over 2 months from August 2018 to October 2018. From this data set of tweets, a set of 4000 tweets was selected, and each tweet was manually annotated for relevance (vape relevant or not), commercial nature (commercial or not), and sentiment (provape or not). Using the annotated data, we derived traditional classifiers that included logistic regression, random forest, linear support vector machine, and multinomial naive Bayes. In addition, using the annotated data set and a larger unannotated data set of tweets, we derived deep learning classifiers that included a convolutional neural network (CNN), long short-term memory (LSTM) network, LSTM-CNN network, and bidirectional LSTM (BiLSTM) network. The unannotated tweet data were used to derive word vectors that deep learning classifiers can leverage to improve performance.

**Results:**

LSTM-CNN performed the best with the highest area under the receiver operating characteristic curve (AUC) of 0.96 (95% CI 0.93-0.98) for relevance, all deep learning classifiers including LSTM-CNN performed better than the traditional classifiers with an AUC of 0.99 (95% CI 0.98-0.99) for distinguishing commercial from noncommercial tweets, and BiLSTM performed the best with an AUC of 0.83 (95% CI 0.78-0.89) for provape sentiment. Overall, LSTM-CNN performed the best across all 3 classification tasks.

**Conclusions:**

We derived and evaluated traditional machine learning and deep learning classifiers to identify vaping-related relevant, commercial, and provape tweets. Overall, deep learning classifiers such as LSTM-CNN had superior performance and had the added advantage of requiring no preprocessing. The performance of these classifiers supports the development of a vaping surveillance system.

## Introduction

### Background

Machine learning methods provide a valuable framework for systematic and automated processing and analysis of data on social media platforms such as Twitter for developing surveillance systems with application to public health. The continuous generation of an enormous amount of content by a vast number of users allows for efficient real-time monitoring of sources of information and user sentiment if it can be automated. Furthermore, such monitoring can lead to the discovery of emergent patterns of information flow and changes in sentiments that may occur in response to public health and policy interventions. In this study, we derived and evaluated traditional machine learning and deep learning classifiers that can be used to build a Twitter-based surveillance system to identify and monitor vaping-related content and sentiments.

### Vaping and Public Health

Vaping is the inhalation of aerosols that often contain nicotine combined with flavorings where the aerosols are delivered through electronic delivery systems known as electronic cigarettes (e-cigarettes) or electronic vaporizers. Evidence suggests that vaping is safer than smoking tobacco and can help with successful smoking cessation [1]. However, emerging research indicates that vaping may cause cardiovascular and respiratory diseases and may pose health hazards from secondhand aerosol exposure [2]. More recently, vaping has been associated with e-cigarette or vaping product use–associated lung injury, which has caused hospitalization and even death [3,4]. There is a rising concern that vaping increases addiction among nonsmokers, especially adolescents [5], and many are unaware of the addictive potential until after they become nicotine dependent [6]. Thus, there is a strong need to measure and understand the risks, sentiments, and behavior related to vaping.

### Surveillance Using Twitter

Twitter is a popular social media platform that is widely used by adolescents, young adults, and racial and ethnic minorities, all of whom are disproportionately affected by vaping [7-9]. Communication on Twitter is by short succinct messages, called tweets, which are limited to 280 characters. Twitter is an open platform that enables users to see information and messages from other public users without special permission. This results in high potential exposure to each tweet, which enables systematic assessment by investigators. Furthermore, tweets heavily use hashtags (eg, #vapelife) as searchable text, which allows users to click on a linked word or phrase and navigate to other mentions of it [10]. These factors make Twitter a relevant, valuable, and feasible social media platform to study.

Infoveillance is the application of surveillance methods to internet-related and other electronic content to inform public health and public policy. Traditional surveys around attitudes and beliefs are too slow to optimally capture rapid changes. Infoveillance methods that use web-based data streams have proven to be more effective for several areas of public health. Investigators have used Twitter data for the infoveillance of topics such as pharmacovigilance, vaccine information, and tracking health conditions [11-13]. For example, such data have been useful in characterizing outbreaks of food-related illness and influenza, factors surrounding prescription drug abuse [14], adverse drug events [15], sentiment toward the use of tobacco [16,17], and use of alcohol [18].

### Objective

Our immediate objective was to derive and evaluate machine learning classifiers that can form the basis of a Twitter-based surveillance system that is focused on vaping-related tweets. Our ultimate goal is to use a surveillance system to assess key factors such as sentiment, marketing, procurement, health effects, and policy that will provide unique perspectives related to vaping. Furthermore, we plan to characterize changes over time in the volume of messaging related to vaping and other vaping-related characteristics of interest [19,20]. Leveraging Twitter as a complement to traditional surveillance will allow for real-time identification of changes that can be used by public health practitioners. For example, when positive sentiment toward vaping rises, practitioners may be able to determine reasons for this and respond accordingly. Similarly, when there is a notable spike in misinformation about vaping and health effects, they will be able to act immediately to correct this information. As a step toward the development of a Twitter-based vaping surveillance system, we derived machine learning classifiers to automatically identify tweets that are vaping-related, are noncommercial, and express provape sentiments. Using a data set of manually annotated tweets and a larger data set of unannotated tweets, we derived and evaluated traditional machine learning and deep learning classifiers.

### Related Work

Natural language processing, classification, and sentiment analysis of Twitter data are more taxing than other kinds of text because of the limited length of the tweets. As tweets are limited to 280 characters and the language used is informal, the messages are interspersed with abbreviations, slang, Twitter-specific terms such as usernames and hashtags, and URLs.

Several investigators have derived classifiers using Twitter data in the context of vaping. For example, Han and Kavuluru [21] implemented support vector machines, logistic regression, and convolutional neural networks to identify marketing and nonmarketing e-cigarette tweets. Myslin et al [17] and Cole-Lewis et al [22] annotated tobacco-related tweets and derived several machine learning classifiers to predict sentiment. Huang et al [23] analyzed tweets using classifiers and found that tweets related to e-cigarettes were about 90% commercial and about 10% mentioned smoking cessation. Resende and Culotta [24] derived a sentiment classifier for e-cigarette–related tweets that identified positive and negative tweets with 96% and 70% precision, respectively.

Compared with prior work, the main contributions of this paper are (1) exploration of a large range of classifiers, including deep learning classifiers; and (2) analysis of highly relevant features in classifiers using an algorithm that provides a unified approach to explain the output of any classifier.

## Methods

### Data Collection

Primary data were collected from the Twitter application programming interface using the open-source, real-time infoveillance of Twitter health messages (RITHM) software [19]. RITHM allows for the real-time collection of all publicly available tweets matching a specified set of keywords. We identified and collected all tweets that matched one or more keywords that are indicative of vaping-related tweets. The keywords that we used for data collection included *vape, vapes, vaper, vapers, vaping, juul, juuls,* and *juuling*. The vaping-related keywords are based on previous Twitter research [10], and, in particular, we included keywords to identify the highly popular JUUL e-cigarette [6].

#### Data Set for Annotation

We continuously collected all publicly available tweets that matched vaping-related keywords over 2 months from August 17, 2018, to October 19, 2018. This resulted in a data set of 1,892,722 tweets. From this data set, we removed *retweets* (rebroadcasted messages without original content), and from the remaining original 810,600 tweets, we randomly selected a subset of 4000 tweets for manual double coding and adjudication. The removal of retweets and the random selection ensured that the tweet content was lexically diverse and sufficiently representative of tweets related to vaping. This particular period was chosen as it also included salient health policy events related to vaping. In particular, the US Food and Drug Administration (FDA) sent warning letters to retailers and manufacturers (September 12, 2018) and seized documents from JUUL headquarters (October 05, 2018). In previous studies, data sets of 4000 to 7000 tweets have been adequate for the derivation of classifiers [17,25,26].

#### Unannotated Data Set for Deriving Word Vectors

Word vectors, also known as word embeddings, are derived from a large data set of text to capture semantic and syntactic similarity and context of each word as a vector of real numbers. Word vectors have become popular because they can improve the performance of deep learning classifiers and can reduce the volume of annotations that are needed. Word vectors have the advantage that they do not require annotations; instead, they leverage a large amount of unannotated data.

We continuously collected all publicly available tweets that matched vaping-related keywords over 7 months from January 01, 2018 to July 31, 2018. This resulted in a data set of 4,078,343 tweets, and from this data set, we removed retweets to obtain a set of 1,899,851 original tweets. We used this set to derive word vectors for deep learning. The period of data selected for word vectors represents 7 months of continuous data collection and provided a sufficiently large set of tweets for deriving word vectors and simultaneously ensuring that relevant context from the tweets, in terms of language and topical diversity, is captured in the word vectors. The period of data selected for word vectors was before the period of data selected for annotations, with no overlap, as a part of the annotated set was used for the evaluation of the classifiers.

### Annotation

**Figure 1:**
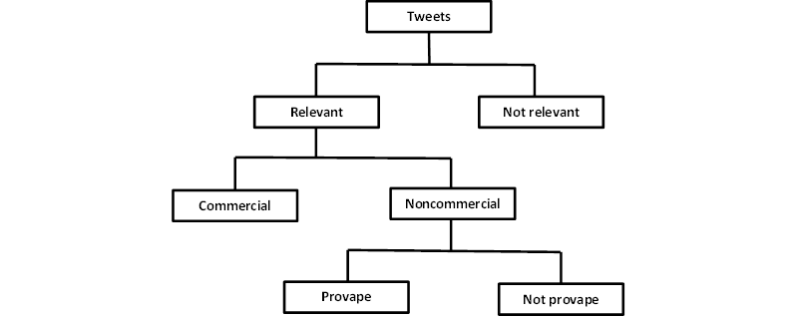
A hierarchical annotation scheme for vaping-related tweets.

We developed a three-level hierarchical annotation schema, as shown in Figure 1. Descriptions of the labels used for annotation are provided in Table 1. The annotation procedure consisted of first annotating a tweet as vape relevant or not based on the content. A relevant tweet was further annotated as commercial or noncommercial, and a noncommercial tweet was further annotated for provape or not provape sentiments. A similar three-level hierarchical annotation schema has been used for annotating vaccination-related tweets. At the first level, a tweet is annotated as relevant or not; at the next level, only a relevant tweet is annotated as positive, negative, or neutral; and at the final level, only a negative tweet is annotated based on safety, efficacy, cost, etc [25,26]. A hierarchical annotation schema has the advantage that all tweets need not be annotated on all possible levels, thus allowing for a reduction in annotation effort. For example, nonrelevant tweets need not be annotated further, and relevant and commercial tweets need not be annotated further.

Trained annotators independently annotated 4000 tweets in batches of 100 to 200 and adjudicated annotation disagreements in the presence of a supervising investigator. Annotators considered tweet content that included both primary and secondary text (ie, quoted tweets within primary tweets). Furthermore, annotators had access to Twitter’s native platform, where they could review the context of potentially confusing content. Cohen κ coefficient was used to assess interrater agreement [27] before adjudication and at regular intervals throughout the process. Initial κ coefficients were relatively modest (eg, κ=0.54 for relevance), but improved as annotators gained familiarity with the data and the domain. The κ coefficients for the final round of annotation (n=100) were 0.71 for relevance, 0.89 for commercial, and 0.70 for provape. Fully adjudicated annotations and tweet content including metadata were used for machine learning.

**Table 1:** Descriptions of labels used for annotating vaping-related tweets.

Labels	Descriptions
Relevant	Is the tweet in English and related to the vaping topic at hand (eg, vape use or users, vaping devices, or products)?
Not relevant	Tweets categorized as not relevant were typically in non-English or had referenced vaping cannabis products specifically, such as: “Teens are smoking, vaping and eating cannabis” “What if I vape weed?”
Commercial	Is the tweet selling, marketing, or advertising vaping products?
Noncommercial	Includes tweets that demonstrate favorability toward a product but do not directly advocate for purchasing it.
Provape	Is vaping associated with positive emotions or contexts? Such as: The tweet author is currently using, has recently used, or intends to use a vape product. The tweet author indicates acceptance of others’ vaping or favorability toward others’ positive perspectives of vaping. The tweet author mentions vaping in association with other positive aspects of society or popular culture (eg, partying, sexuality, popularity, and attractiveness).
Not provape	Includes tweets that are antivape, neutral or fact based, or without subjective judgment about positive or acceptable aspects of vaping.

### Machine Learning

In this section, we describe the steps in machine learning that consist of preprocessing, derivation of features, and training of classifiers.

#### Preprocessing and Vector Representation for Traditional Classifiers

Twitter data consist of tweet metadata and tweet content. Metadata includes information related to the user’s profile (such as location, number of followers, number of friends, and tweeting frequency), information related to a tweet’s status (such as the location of the tweet), media object contained in the tweet (such as audio, video, and image), and if the tweet was in reply to another tweet. As tweets are restricted to 280 characters, their content has, in addition to the standard text, abbreviations, usernames (that are annotated with the @), hashtags (topic tags annotated with the *#*), Unicode characters, URLs (typically shortened pseudorandom short URLs), and emojis (icons used to express an idea or emotion). Before preprocessing, we replaced usernames, hashtags, Unicode characters, and URLs with the textual placeholders _mention_, _hashtag_, _unicode_, and _url_, respectively. We also translated emojis into textual descriptions for better interpretability. This standardized text representation of tweets ensured that the preprocessing pipeline needed to handle only text.

The preprocessing pipeline consisted of 10 steps, including removal of textual placeholders (for usernames, hashtags, Unicode characters, and URLs), removal of textual descriptions of emojis, expansion of negations, removal of punctuation and digits, negation marking, normalization, stemming, removal of stopwords, and conversion to lowercase (Table 2).

After preprocessing, we created 2 types of tweet representations that are useful for machine learning. In the first representation, called the vector count representation, we identify unique words in the tweet data set and represent each tweet with a vector of numbers, where a number denotes the frequency (count) of the occurrence of a unique word in the tweet. Thus, each tweet is represented by a vector that contains as many counts as the number of unique words. We also investigated an alternative vector representation called frequency-inverse document frequency (TF-IDF) where the number assigned to a word in the vector depends not only on its frequency in a tweet but also on its frequency in the entire data set. In this representation, words that occur in the majority of the tweets are considered to be of lower importance than words that occur more rarely. As preliminary results did not demonstrate improved performance with the TF-IDF representation, we did not perform extensive experiments with this representation.

Rather than applying the same set of preprocessing steps to every classifier, we searched all possible combinations of the 10 preprocessing steps for each classifier and identified the optimal set of preprocessing steps that gave the best classifier performance (Table 2).

**Table 2:** Description of preprocessing steps and options used in traditional classifiers.

Preprocessing steps	Descriptions	Options^a^
placeholder_remove	Remove textual placeholders such as _mention_, _hashtag_, _unicode_, and _url_	True, false
emoji_remove	Remove textual descriptions that denote emojis	True, false
negation_expand	Expand negative contractions, for example, “don’t” is expanded to “do not” and “can’t” is expanded to “cannot”	True, false
punctuation_remove	Remove all punctuation symbols	True, false
digits_remove	Remove all numeric digits (0-9)	True, false
negation_mark	Mark words that occur between a negation trigger and a punctuation mark with the NEG prefix [28]	True, false
normalize	Reduce to 2 characters all consecutive characters that appear more than twice, for example, “happppy” is reduced to “happy”	True, false
stemming	Reduce inflection in words (eg, troubled, troubles) to their root form (eg, trouble) using the Porter Stemmer [29]	True, false
stopwords_remove	Remove common words such as “the,” “a,” “on,” “is,” and “all” that are listed in the Natural Language Toolkit English stop words list [30]	True, false
lowercase	Change the case of all characters to lowercase	True, false

^a^If the option for a step is set to *true*, the corresponding preprocessing step will be applied in the preprocessing pipeline; if the option is set to *false*, the corresponding preprocessing step will be skipped in the pipeline.

#### Preprocessing and Vector Representation for Deep Learning Classifiers

For the deep learning classifiers, we used 2 alternative preprocessing methods: (1) a fixed preprocessing pipeline and (2) no preprocessing. The fixed preprocessing pipeline consisted of the following 5 steps (out of the possible 10 steps listed in Table 2): removal of textual placeholders, expansion of negations, removal of punctuation and digits, and conversion to lowercase. In contrast to vector count representation, which is used in traditional classifiers where a tweet is denoted by a vector of counts, in the deep learning classifiers, each word in a tweet is denoted by a word vector as described next, and each tweet is denoted by a vector of word vectors.

#### Word Vectors

Word vectors are derived from large unannotated tweet data (or other types of text data) and are increasingly used in deep learning classifiers. A word vector represents a word (not an entire tweet as in vector count representation) as a vector of numbers such that 2 words are considered to be similar in meaning if their vectors are close to each other mathematically. Word vectors capture the meaning and usage of words and are derived from patterns of how words co-occur in a large data set of tweets.

We investigated the performance of word vectors from 2 types of tweet data. First, we used word vectors that are derived from a large data set of tweets of all kinds; we call these vectors general or nondomain-specific word vectors. For general word vectors, we downloaded the 200-dimension Global Vectors for Word Representation (GloVe) word vectors. The GloVe vectors were derived from 2 billion tweets of all kinds, and each word was represented by a vector of size 200 [31]. Second, we used word vectors from a large data set of vaping-related tweets; we call these vectors vaping-related word vectors. We created vaping-related word vectors from a data set of tweets that were collected over 7 months from January 01, 2018 to July 31, 2018 using the vaping-related keywords. This data set contained 1,899,851 original tweets, and we used the Word2Vec algorithm [32] to derive 300-dimension word vectors (additional settings for the Word2Vec algorithm included a window size of 2 and 30 epochs).

#### Machine Learning Methods

We derived and evaluated 2 families of classifiers. The traditional classifiers included logistic regression (LR), random forest (RF), linear support vector machine (SVM), and multinomial naive Bayes (NB), and we used the implementations of these classifiers in scikit-learn version 0.23.1 [33]. The deep learning classifiers included convolutional neural network (CNN), long short-term memory (LSTM) network, combined LSTM and CNN (LSTM-CNN), and bidirectional LSTM (BiLSTM) network, and we used the implementations of these classifiers in Keras version 2.2.4 [34].

In contrast to traditional classifiers, CNNs automatically select words in tweets that are relevant. The LSTM network is a type of neural network that captures patterns of words in tweets. Conventional LSTM networks capture patterns in a single direction, from left to right, whereas BiLSTM networks capture patterns in both directions, from left to right and from right to left. Both LSTM and BiLSTM have demonstrated good performance on social media data [35,36], and compared with CNNs, they can handle the variable lengths of tweets. The LSTM-CNN networks combine the advantages of CNNs and LSTM networks.

We derived and evaluated separate classifiers for 3 different tasks, that is, to identify which tweets are relevant, are noncommercial, and contain provape sentiment. For these tasks, the 3 binary targets and their corresponding values are (1) relevance: relevant (positive value) versus nonrelevant (negative value), (2) commercial: commercial (positive value) versus noncommercial (negative value), and (3) sentiment: provape (positive value) versus not provape (negative value).

#### Experimental Methods

From the annotated data set of 4000 tweets, we created 3 data sets to predict relevance, commercial, and sentiment that contained 4000, 3011, and 2175 tweets, respectively (Table 3). Each data set was randomly split into training and test sets (90:10 splits) such that the sets contained the same proportion of positive targets. A total of 3600, 2709, and 1957 tweets were used in the training data sets to derive relevance, commercial, and sentiment classifiers, respectively (Table 3). We used the training set to derive the best classifier (including the selection of hyperparameters if needed) for each type of classifier. The test data sets that were used to evaluate the relevance, commercial, and sentiment classifiers included 400, 302, and 218 tweets, respectively (Table 3).

Table 4 shows the traditional classifiers with parameter settings that we used in our experiments, and Table 5 shows the parameter settings of the deep learning classifiers that we used in our experiments.

**Table 3:** Description of training and test data sets.

Targets	Total number of tweets, n (%)	Number of tweets with positive target, n (%)	Number of tweets with negative target, n (%)
Relevance	⁠ Total: 4000 (100) Training: 3600 (100) Test: 400 (100)	Relevant Total: 3011 (75.28) Training: 2709 (75.25) Test: 302 (75.5)	Nonrelevant Total: 989 (24.72) Training: 891 (24.75) Test: 98 (24.5)
Commercial	⁠ Total: 3011 (100) Training: 2709 (100) Test: 302 (100)	Noncommercial Total: 2175 (72.24) Training: 1957 (72.24) Test: 218 (72.2)	Commercial Total: 836 (27.76) Training: 752 (27.86) Test: 84 (27.8)
Sentiment	⁠ Total: 2175 (100) Training: 1957 (100) Test: 218 (100)	Provape Total: 1357 (62.39) Training: 1221 (62.39) Test: 136 (62.4)	Not provape Total: 818 (37.61) Training: 736 (37.61) Test: 82 (37.6)

**Table 4:** Description of traditional classifiers and parameter settings used in the experiments (the same parameter settings were used for the following 3 targets: relevance, commercial, and sentiment).

Classifiers	Scikit-learn functions (version)	Parameter values
Logistic regression	sklearn.linear_model.LogisticRegression (0.20.3)	All default values except C=0.001
Random forest	sklearn.ensemble.RandomForestClassifier (0.20.3)	All default values except max_features=“sqrt”
Support vector machine	sklearn.linear_model.SGDClassifier (0.20.3)	All default values except α=.01
Naive Bayes	sklearn.naive_bayes.MultinomialNB (0.20.3)	All default values

**Table 5:** Description of deep learning classifiers, target, and parameter settings used in the experiments.

Deep learning classifiers		Targets	Parameter values
**Vaping-related word vectors**			
	CNN^a^	Relevance	max_features: 166,395, embed_size: 300, max_len: 75, optimizer: rmsprop, filters: 100, kernel_size: 1, epochs: 5, batch_size: 16
	LSTM^b^	Relevance	max_features: 166,395, embed_size: 300, max_len: 75, optimizer: adam, epochs: 10, batch_size: 16
	LSTM-CNN	Relevance	max_features: 166,395, embed_size: 300, max_len: 75, optimizer: adam, filters: 50, kernel_size: 2, epochs: 10, batch_size: 16
	BiLSTM^c^	Relevance	max_features: 166,395, embed_size: 300, max_len: 75, optimizer: adam, epochs: 10, batch_size: 16
	CNN	Commercial	max_features: 166,395, embed_size: 300, max_len: 75, optimizer: adam, filters: 100, kernel_size: 2, epochs: 10, batch_size: 16
	LSTM	Commercial	max_features: 166,395, embed_size: 300, max_len: 75, optimizer: rmsprop, epochs: 5, batch_size: 32
	LSTM-CNN	Commercial	max_features: 166,395, embed_size: 300, max_len: 75, optimizer: rmsprop, filters: 75, kernel_size: 2, epochs: 5, batch_size: 16
	BiLSTM	Commercial	max_features: 166,395, embed_size: 300, max_len: 75, optimizer: adam, epochs: 5, batch_size: 64
	CNN	Sentiment	max_features: 166,395, embed_size: 300, max_len: 75, optimizer: rmsprop, filters: 100, kernel_size: 2, epochs: 10, batch_size: 32
	LSTM	Sentiment	max_features: 166,395, embed_size: 300, max_len: 75, optimizer: adam, epochs: 5, batch_size: 64
	LSTM-CNN	Sentiment	max_features: 166,395, embed_size: 300, max_len: 75, optimizer: adam, filters: 75, kernel_size: 3, epochs: 5, batch_size: 64
	BiLSTM	Sentiment	max_features: 166,395, embed_size: 300, max_len: 75, optimizer: rmsprop, epochs: 5, batch_size: 32
**Global Vectors for Word Representation** **word vectors**			
	CNN	Relevance	max_features: 15,890, embed_size: 200, max_len: 75, optimizer: adam, filters: 100, kernel_size: 2, epochs: 10, batch_size: 16
	LSTM	Relevance	max_features: 15,890, embed_size: 200, max_len: 75, optimizer: adam, epochs: 5, batch_size: 32
	LSTM-CNN	Relevance	max_features: 15,890, embed_size: 200, max_len: 75, optimizer: adam, filters: 50, kernel_size: 2, epochs: 10, batch_size: 16
	BiLSTM	Relevance	max_features: 15,890, embed_size: 200, max_len: 75, optimizer: adam, epochs: 5, batch_size: 64
	CNN	Commercial	max_features: 10,842, embed_size: 200, max_len: 75, optimizer: rmsprop, filters: 50, kernel_size: 2, epochs: 5, batch_size: 16
	LSTM	Commercial	max_features: 10,842, embed_size: 200, max_len: 75, optimizer: adam, epochs: 5, batch_size: 16
	LSTM-CNN	Commercial	max_features: 10,842, embed_size: 200, max_len: 75, optimizer: adam, filters: 75, kernel_size: 2, epochs: 5, batch_size: 32
	BiLSTM	Commercial	max_features: 10,842, embed_size: 200, max_len: 75, optimizer: adam, epochs: 5, batch_size: 64
	CNN	Sentiment	max_features: 7979, embed_size: 200, max_len: 75, optimizer: rmsprop, filters: 100, kernel_size: 3, epochs: 5, batch_size: 64
	LSTM	Sentiment	max_features: 7979, embed_size: 200, max_len: 75, optimizer: adam, epochs: 5, batch_size: 32
	LSTM-CNN	Sentiment	max_features: 7979, embed_size: 200, max_len: 75, optimizer: rmsprop, filters: 75, kernel_size: 1, epochs: 10, batch_size: 64
	BiLSTM	Sentiment	max_features: 7979, embed_size: 200, max_len: 75, optimizer: adam, epochs: 5, batch_size: 32

^a^CNN: convolutional neural network.

^b^LSTM: long short-term memory.

^c^BiLSTM: bidirectional long short-term memory.

#### Evaluation of Classifier Performance

We assessed the performance of the classifiers with the area under the receiver operating characteristic curve (AUC), precision, recall, and F1 scores. The AUC is a measure of discrimination, that is, how well a classifier differentiates between the positive and negative tweets, and larger values indicate better performance. Precision is the number of correctly classified positive tweets divided by the number of all positive tweets returned by the classifier, and recall is the number of correctly classified positive tweets divided by the number of all positive tweets. The F1 score is the harmonic average of the precision and recall; the F1 score achieves the best value at 1 when both precision and recall are perfect and the worst value at 0.

#### Evaluation of Relevance

To identify relevant words (features) in each classifier, we applied SHapley Additive exPlanations (SHAP), which is an algorithm for interpreting the relevance of features used in classifiers [37]. SHAP assigns each feature an average relevance value based on predictions on a data set. We examined the top 10 ranked features for each classifier.

## Results

### Proportions of Tweet Categories in the Annotated Data Set

In the annotated data set, 75.28% were relevant to vaping, and of the vaping-relevant tweets, 72.24% were of a noncommercial nature. Of the noncommercial vaping-relevant tweets, 62.39% contained provape sentiments.

### Performance of Classifiers

#### Relevance Classifiers

Application of traditional classifiers yielded AUC values of 0.84 to 0.95, application of deep learning classifiers with vaping-related word vectors yielded AUC values of 0.90 to 0.93, and application of deep learning classifiers with GloVe word vectors yielded AUC values of 0.93 to 0.96. LR had the highest recall, whereas RF and the deep learning classifiers with GloVe word vectors had the highest F1 value. LSTM-CNN with GloVe word vectors performed the best overall with the highest AUC and precision values.

#### Commercial Classifiers

Overall, the AUC values were similar across all classifiers. Application of traditional classifiers yielded AUC values of 0.96 to 0.98, application of deep learning classifiers with vaping-related word vectors yielded AUC values of 0.97 to 0.98, and application of deep learning classifiers with GloVe word vectors yielded AUC values of 0.99. LSTM-CNN and BiLSTM with GloVe word vectors performed the best overall with the highest AUC, precision, recall, and F1 values.

#### Sentiment Classifiers

Application of traditional classifiers yielded AUC values of 0.69 to 0.78, application of deep learning classifiers with vaping-related word vectors yielded AUC values of 0.74 to 0.75, and application of deep learning classifiers with GloVe word vectors yielded AUC values of 0.78 to 0.83. BiLSTM and LSTM-CNN with GloVe word vectors performed the best overall with the highest AUC, precision, and F1 values.

#### Preprocessing

Our experiments showed that some traditional classifiers performed best with minimal preprocessing compared with others. LR and NB did not use any of the 10 preprocessing steps for any of the 3 targets (Multimedia Appendix 1). On the other hand, RF and SVM used 5 preprocessing steps on average (Multimedia Appendix 1). The deep learning classifiers performed better with no preprocessing compared with the fixed preprocessing pipeline. Furthermore, in addition to the standard text in tweets, information such as URLs, usernames, hashtags, and Unicode characters was found to be important and was included in most of the classifiers.

### Feature Relevance

We applied the SHAP algorithm to the 12 classifiers for each target (corresponding to the classifiers in Tables 6, 7, and 8) to generate 10 top-ranked features. The feature relevance plots for each classifier and target are shown in Multimedia Appendix 1. The word *vape* and its variations *vapes*, *vaping*, or *vapelife* appear in the 10 top-ranked features in all classifiers except RF relevance and commercial classifiers. Several textual placeholders appear in traditional classifiers, whereas several Unicode characters representing emojis appear in the deep learning classifiers. Interestingly, common simple words such as *we*, *as*, *was*, and *no* appear in many classifiers.

**Table 6:** Performance of relevance classifiers.

Classifiers	Area under the receiver operating characteristic curve (95% CI)	Precision	Recall	F1
Logistic regression	0.84 (0.78-0.89)	0.80	1.00	0.92
Random forest	0.95 (0.93-0.98)	0.93	0.97	*0.98*
Support vector machine	0.92 (0.88-0.96)	0.91	0.97	0.95
Naive Bayes	0.88 (0.83-0.93)	0.88	0.99	0.93
CNN^a^ (vaping-related word vectors)	0.94 (0.91-0.97)	0.90	0.97	0.98
LSTM^b^ (vaping-related word vectors)	0.91 (0.88-0.95)	0.89	0.98	0.96
LSTM-CNN (vaping-related word vectors)	0.89 (0.85-0.93)	0.93	0.87	0.95
BiLSTM^c^ (vaping-related word vectors)	0.89 (0.85-0.94)	0.90	0.96	0.94
CNN (GloVe^d^ word vectors)	0.95 (0.92-0.97)	0.93	0.95	0.98
LSTM (GloVe word vectors)	0.95 (0.92-0.98)	0.95	0.95	0.98
LSTM-CNN (GloVe word vectors)	0.96 (0.93-0.98)	0.96	0.93	0.98
BiLSTM (GloVe word vectors)	0.95 (0.93-0.98)	0.92	0.96	0.98

^a^CNN: convolutional neural network.

^b^LSTM: long short-term memory.

^c^BiLSTM: bidirectional long short-term memory.

^d^GloVe: Global Vectors for Word Representation.

**Table 7:** Performance of commercial classifiers.

Classifiers	Area under the receiver operating characteristic curve (95% CI)	Precision	Recall	F1
Logistic regression	0.98 (0.95-0.99)	0.93	0.83	0.96
Random forest	0.97 (0.96-0.99)	0.95	0.82	0.97
Support vector machine	0.98 (0.91-0.99)	0.92	0.86	0.92
Naive Bayes	0.96 (0.94-0.99)	0.83	0.89	0.92
CNN^a^ (vaping-related word vectors)	0.98 (0.96-0.99)	0.93	0.75	0.94
LSTM^b^ (vaping-related word vectors)	0.97 (0.95-0.99)	0.88	0.81	0.94
LSTM-CNN (vaping-related word vectors)	0.97 (0.94-0.99)	0.92	0.85	0.94
BiLSTM^c^ (vaping-related word vectors)	0.98 (0.96-0.99)	0.84	0.87	0.95
CNN (GloVe^d^ word vectors)	0.99 (0.98-0.99)	0.93	0.89	0.98
LSTM (GloVe word vectors)	0.99 (0.98-0.99)	0.89	0.94	0.98
LSTM-CNN (GloVe word vectors)	0.99 (0.98-0.99)	0.86	0.96	0.99
BiLSTM (GloVe word vectors)	0.99 (0.98-0.99)	0.97	0.88	0.98

^a^CNN: convolutional neural network.

^b^LSTM: long short-term memory.

^c^BiLSTM: bidirectional long short-term memory.

^d^GloVe: Global Vectors for Word Representation.

**Table 8:** Performance of sentiment classifiers.

Classifiers	Area under the receiver operating characteristic curve (95% CI)	Precision	Recall	F1
Logistic regression	0.78 (0.71-0.84)	0.73	0.88	0.82
Random forest	0.78 (0.70-0.83)	0.78	0.79	0.82
Support vector machine	0.69 (0.64-0.78)	0.66	0.98	0.75
Naive Bayes	0.75 (0.66-0.82)	0.75	0.79	0.80
CNN^a^ (vaping-related word vectors)	0.74 (0.66-0.81)	0.73	0.85	0.80
LSTM^b^ (vaping-related word vectors)	0.74 (0.69-0.82)	0.75	0.81	0.81
LSTM-CNN (vaping-related word vectors)	0.75 (0.71-0.84)	0.74	0.91	0.83
BiLSTM^c^ (vaping-related word vectors)	0.74 (0.68-0.81)	0.72	0.91	0.82
CNN (GloVe^d^ word vectors)	0.81 (0.75-0.87)	0.72	0.96	0.86
LSTM (GloVe word vectors)	0.78 (0.71-0.84)	0.76	0.82	0.84
LSTM-CNN (GloVe word vectors)	0.80 (0.74-0.86)	0.83	0.84	0.84
BiLSTM (GloVe word vectors)	0.83 (0.78-0.89)	0.79	0.79	0.88

^a^CNN: convolutional neural network.

^b^LSTM: long short-term memory.

^c^BiLSTM: bidirectional long short-term memory.

^d^GloVe: Global Vectors for Word Representation.

## Discussion

### Principal Findings

The relative prevalence of the 3 categories that we annotated in our data set reflects the general level of vaping-related discussions on Twitter. The high proportion of tweets of a commercial nature (72% of vaping-related tweets) reflects the observation that manufacturers of vaping products marketed their products heavily on Twitter. However, this percentage has likely decreased significantly since the beginning of 2020 because of the introduction of advertising restrictions by federal and state authorities. A high proportion of noncommercial tweets contained provape sentiments (62.39% of noncommercial tweets), suggesting that among Twitter users who post about vaping, the sentiment is overall more positive than negative in our data set, after the exclusion of marketing tweets. This reflects the growing prevalence of vaping, especially among adolescents who post more on Twitter than other age groups [38]. However, as this study used data before the FDA banned a range of flavored e-cigarette cartridges, both vaping and positive sentiments related to vaping may have decreased significantly.

Classifiers that we derived from our data set demonstrated high levels of performance, indicating that currently available machine learning methods can produce high-performing classifiers on a data set of only several thousand annotated tweets. Compared with traditional classifiers, deep learning classifiers had superior performance with AUC values of 0.96, 0.99, and 0.83 for predicting vaping-relevant, commercial, and provape tweets. Furthermore, our results indicate that deep learning classifiers performed the best with no preprocessing and with nondomain-specific GloVe word vectors. A few studies have shown that no preprocessing may provide better performance with Twitter data [39,40]. More generally, additional research is needed to systematically examine alternate preprocessing regimes for Twitter and other types of text data [41]. Although deep learning classifiers are computationally more expensive to derive compared with traditional classifiers, the lack of preprocessing and derivation of domain-specific word vectors offsets the computational cost. Moreover, the application of deep learning classifiers to new Twitter data is as computationally efficient as traditional classifiers.

Analyses of the 10 top-ranked features show that similar features appear across the classifiers. In addition to English terms, emojis and Unicode characters were often identified as useful features. Several common simple terms also appear as important features; these terms may interact with other features rather than being discriminatory on their own.

### Limitations

Our study has several limitations. First, we used a small list of keywords to restrict our data, rather than using the full Twitter feed. As vaping products and their discussions evolve, the list of keywords will likely become stale and will need to be updated. Second, our annotated data set was of moderate size, though the sample size of 4000 tweets was adequate for obtaining classifiers with high performance. Third, the expression of tweet sentiments related to vaping is likely to vary over time [42]. It would be useful to evaluate the performance of the classifiers on data that are obtained from a different period to assess the generalizability of the classifiers over time. Fourth, there may be geographical variation in sentiments regarding vaping [43], and it would be useful to evaluate the performance of the classifiers on data that are obtained from different locations. In future work, we plan to address the limitations of evaluating the classifiers over time and location. Fifth, it is not clear if individuals with certain personality traits make them more predisposed to express positive or negative sentiments [44]. More research is needed to assess the degree to which sentiment reflects variance in psychological traits versus the situational context in which those traits were expressed. Finally, this study uses data before the FDA banned a range of flavored e-cigarette cartridges that were likely to have been popular among frequent Twitter users, such as adolescents. In future work, we plan to derive classifiers from data that were collected after the FDA ban on flavored e-cigarette cartridges.

### Future Surveillance Research

Machine learning classifiers, especially deep learning classifiers, show promising performance over strictly keyword-based approaches for identifying vaping-related tweets and sentiments related to vaping. This observation provides support for the development of a vaping surveillance system. Twitter surveillance can provide relatively inexpensive opportunities for monitoring the evolution of use and sentiment toward vaping and the effects of regulations on the marketing of vaping products. We plan to develop a surveillance system that will apply the classifiers to tweets to produce daily counts of vaping-related tweets, noncommercial tweets, and provape tweets. These daily counts will be used for future behavioral and attitudinal research related to vaping as well as for correlating changes in behavior and attitudes to changes in policy, such as those issued by the FDA. We plan to use the classifiers derived in this study as a basis for comparison with classifiers that we plan to derive from data obtained after the FDA ban to understand whether the ban has altered vaping-related health attitudes and behaviors. Furthermore, we plan to develop methods to infer the age group of the authors of tweets that will enable the daily tracking of vaping and related sentiments in adolescents.

### Conclusions

We derived and evaluated machine learning classifiers to identify vaping-related relevant, commercial, and provape tweets. We developed a hierarchical classification scheme for vaping-related tweets and applied it to a data set of 4000 selected tweets to manually annotate them. We evaluated both traditional machine learning and deep learning classifiers using the annotated data set of 4000 tweets as well as vaping-related word vectors and GloVe word vectors that are derived from large unannotated tweet data sets. Overall, deep learning classifiers such as LSTM-CNN had superior performance and had the added advantage of requiring no preprocessing. These classifiers pave the way for the development of a vaping surveillance system.

## Appendix

Multimedia Appendix 1 Additional information.

## Abbreviations

AUC: area under the receiver operating characteristic curve
BiLSTM: bidirectional long short-term memory
CNN: convolutional neural network
e-cigarettes: electronic cigarettes
FDA: Food and Drug Administration
GloVe: Global Vectors for Word Representation
LR: logistic regression
LSTM: long short-term memory
NB: naive Bayes
RF: random forest
RITHM: real-time infoveillance of Twitter health messages
SHAP: SHapley Additive exPlanations
SVM: support vector machine
TF-IDF: frequency-inverse document frequency
