# Supplementary material for: Machine Learning Classifiers for Twitter Surveillance of Vaping: Comparative Machine Learning Study
Source: J Med Internet Res. 2020 Aug 12;22(8):e17478. doi: 10.2196/17478 (PMC7450367; doi:10.2196/17478)

## Multimedia Appendix 1

**Abbreviations**

BiLSTM: bidirectional long short-term memory

CNN: convolutional neural network

GLoVe: Global Vectors for Word Representation

LR: logistic regression

LSTM: long short-term memory

NB: naïve Bayes

RF: random forest

SVM: support vector machine

**Table 1**. Best performing preprocessing steps and vector representation for the traditional classifiers and parameter settings used in the experiments.

| **Classifiers** | **Targets** | **Best combination of the 10 preprocessing steps** | **Best performing vector representation**  **(n-gram, maximum number of features)** |
| --- | --- | --- | --- |
| LR | relevance | placeholder_remove: False, emoji_remove: False, negation_expand: False, punctuation_remove: False, digits_remove: False, negation_mark: False, normalize: False, stemming: False, stopwords_remove: False, lowercase: False | Bigram, 3200 |
| RF | relevance | placeholder_remove: False, emoji_remove: True, negation_expand: True, punctuation_remove: True, digits_remove: True, negation_mark: True, normalize: True, stemming: False, stopwords_remove: True, lowercase: True | Unigram, 3200 |
| SVM | relevance | placeholder_remove: True, emoji_remove: False, negation_expand: False, punctuation_remove: False, digits_remove: True, negation_mark: True, normalize: True, stemming: True, stopwords_remove: False, lowercase: False | Unigram, 3200 |
| NB | relevance | placeholder_remove: False, emoji_remove: False, negation_expand: False, punctuation_remove: False, digits_remove: False, negation_mark: False, normalize: False, stemming: False, stopwords_remove: False, lowercase: False | Unigram, 3200 |
| LR | commercial | placeholder_remove: False, emoji_remove: False, negation_expand: False, punctuation_remove: False, digits_remove: False, negation_mark: False, normalize: False, stemming: False, stopwords_remove: False, lowercase: False | Unigram, 3200 |
| RF | commercial | placeholder_remove: False, emoji_remove: False, negation_expand: True, punctuation_remove: True, digits_remove: True, negation_mark: True, normalize: False, stemming: True, stopwords_remove: True, lowercase: False | Unigram, 1600 |
| SVM | commercial | placeholder_remove: False, emoji_remove: False, negation_expand: True, punctuation_remove: True, digits_remove: True, negation_mark: True, normalize: False, stemming: False, stopwords_remove: True, lowercase: False | Unigram, 3200 |
| NB | commercial | placeholder_remove: False, emoji_remove: False, negation_expand: False, punctuation_remove: False, digits_remove: False, negation_mark: False, normalize: False, stemming: False, stopwords_remove: False, lowercase: False | Unigram, 3200 |
| LR | sentiment | placeholder_remove: False, emoji_remove: False, negation_expand: False, punctuation_remove: False, digits_remove: False, negation_mark: False, normalize: False, stemming: False, stopwords_remove: False, lowercase: False | Bigram, 3200 |
| RF | sentiment | placeholder_remove: True, emoji_remove: False, negation_expand: True, punctuation_remove: False, digits_remove: True, negation_mark: False, normalize: False, stemming: True, stopwords_remove: False, lowercase: False | Unigram, 800 |
| SVM | sentiment | placeholder_remove: False, emoji_remove: True, negation_expand: False, punctuation_remove: True, digits_remove: True, negation_mark: False, normalize: True, stemming: True, stopwords_remove: True, lowercase: True | Unigram, 3200 |
| NB | sentiment | placeholder_remove: False, emoji_remove: False, negation_expand: False, punctuation_remove: False, digits_remove: False, negation_mark: False, normalize: False, stemming: False, stopwords_remove: False, lowercase: False | Bigram, 3200 |

**Figure 1a.** The magnitude of 10 top-ranked features in traditional classifiers for relevance. Top row: LR and RF; bottom row: SVM and NB.


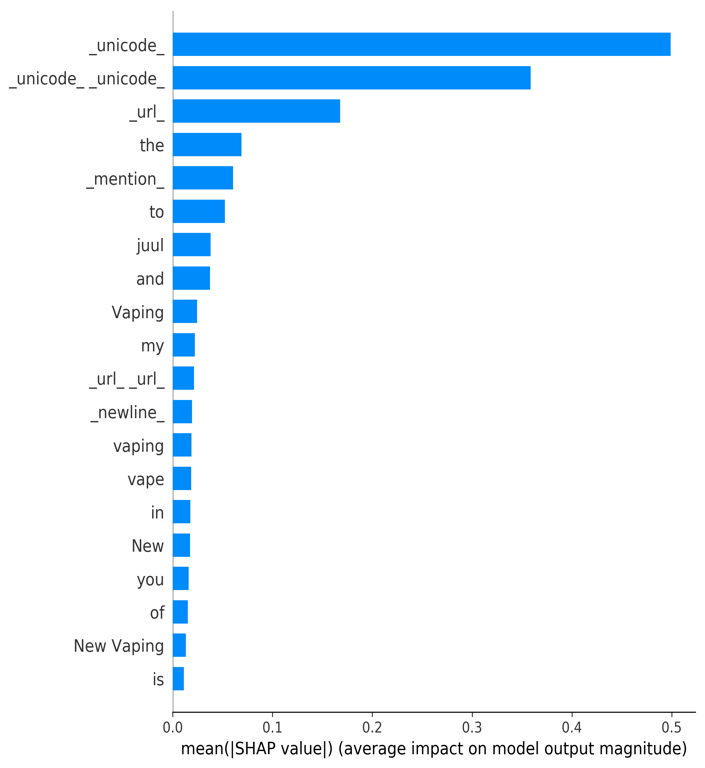

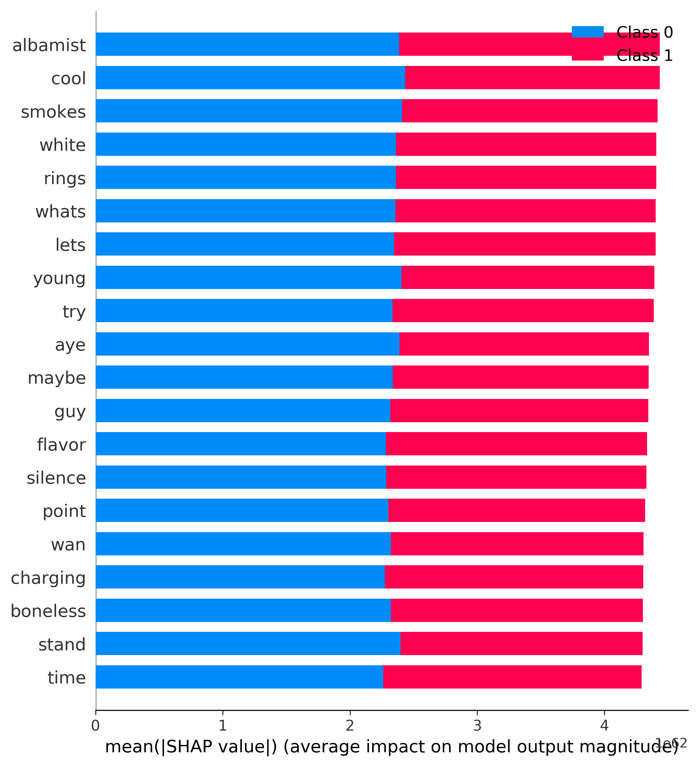


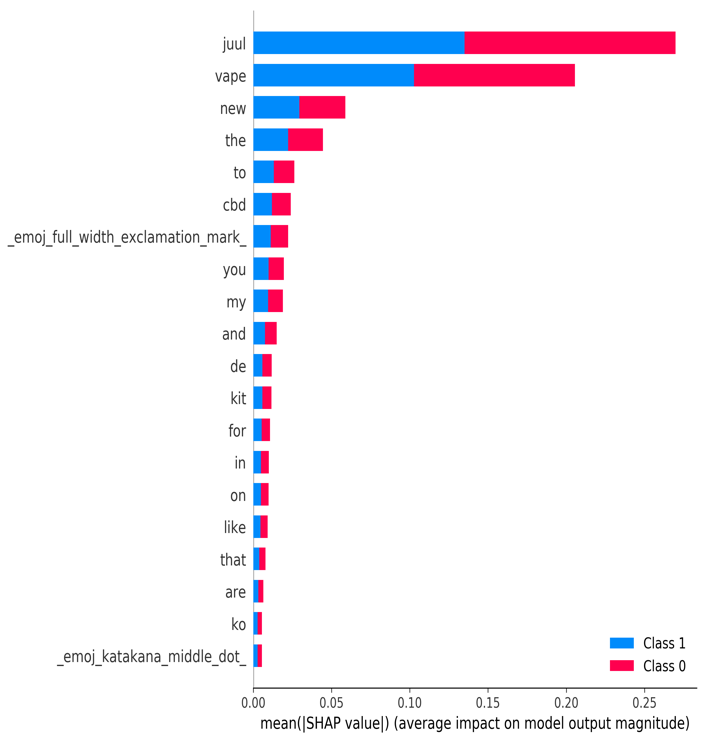

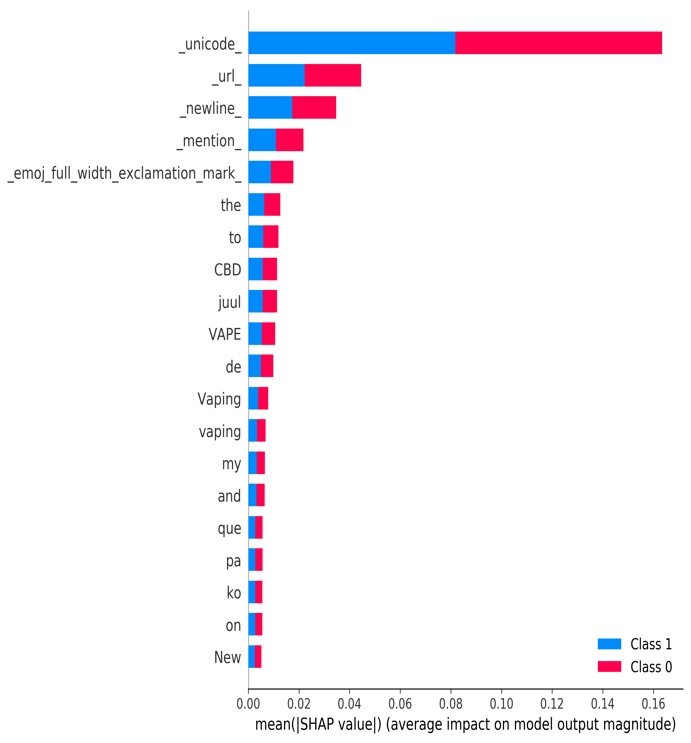


**Figure 1b.** The magnitude of 10 top-ranked features in deep learning classifiers with vaping-related word vectors for relevance. Top row: CNN and LSTM; bottom row: CNN-LSTM and BiLSTM.


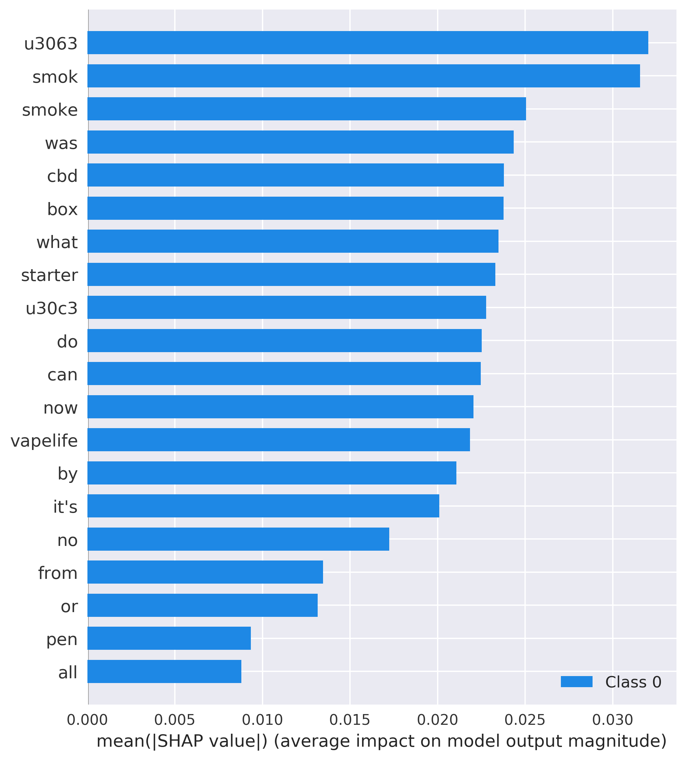

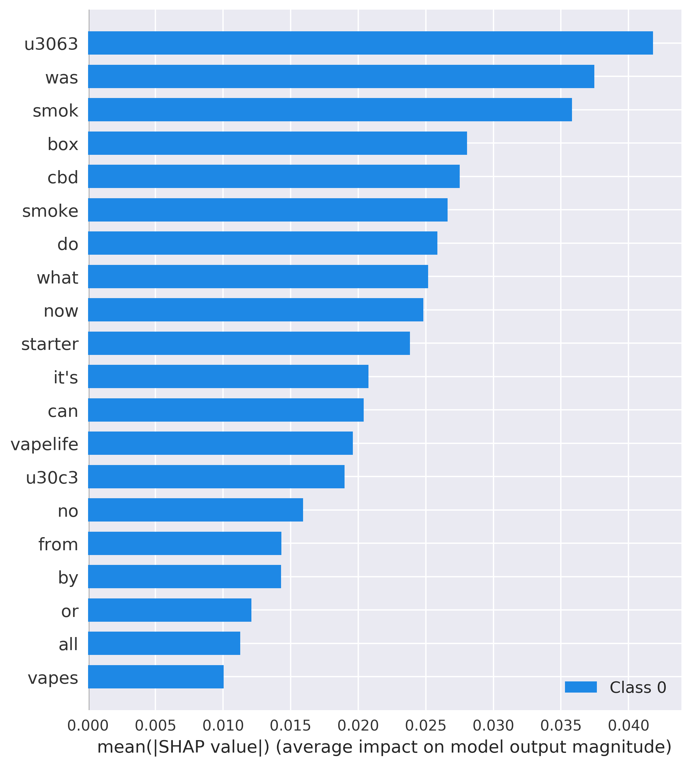


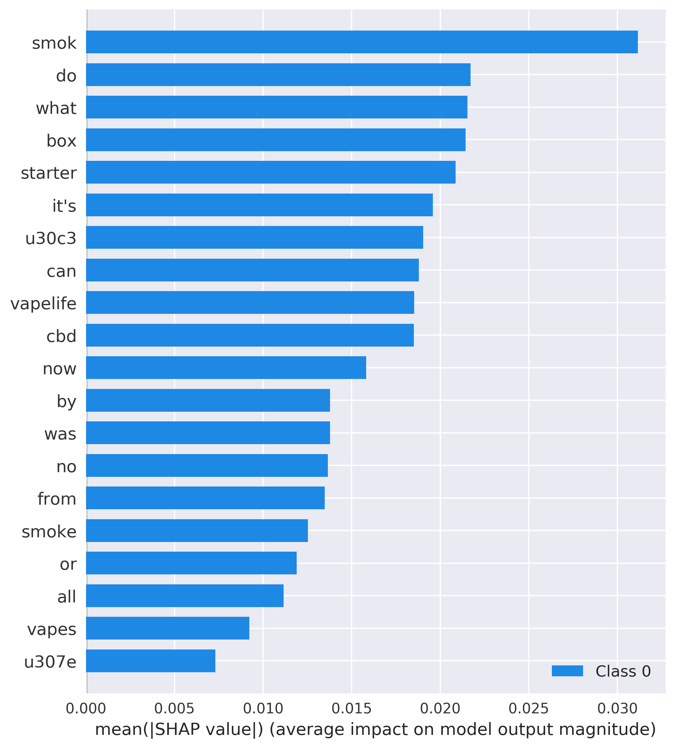

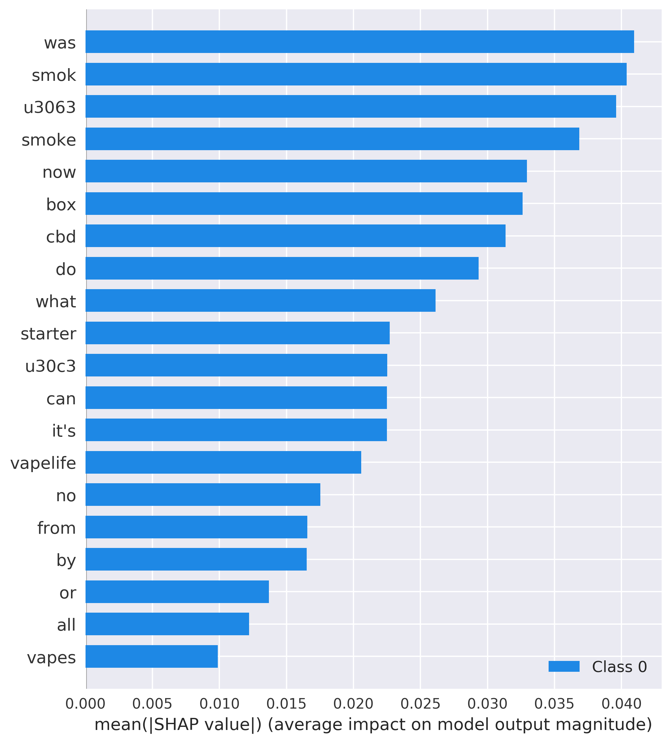


**Figure 1c.** The magnitude of 10 top-ranked features in deep learning classifiers with GloVe word vectors for relevance. Top row: CNN and LSTM; bottom row: CNN-LSTM and BiLSTM. (u3063, u30c3, u307e are Unicode characters)


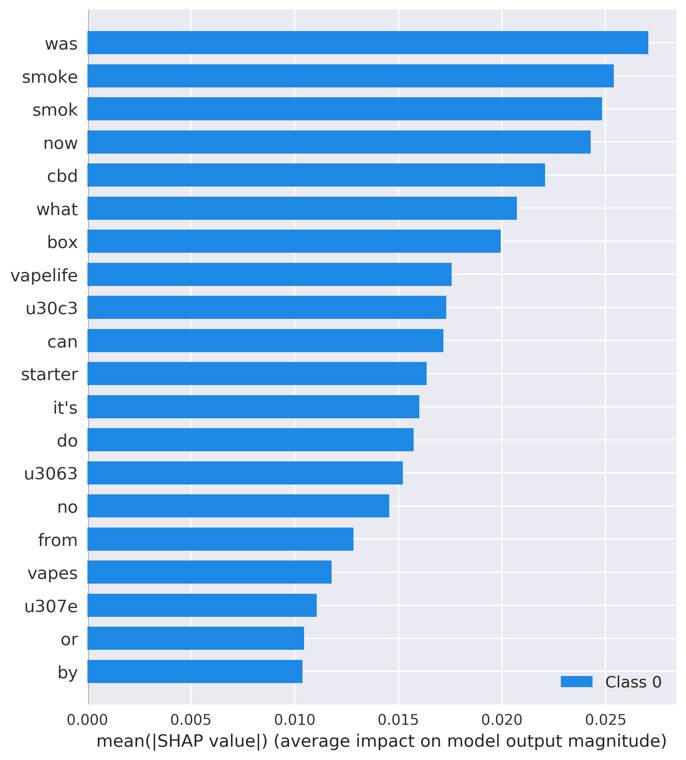

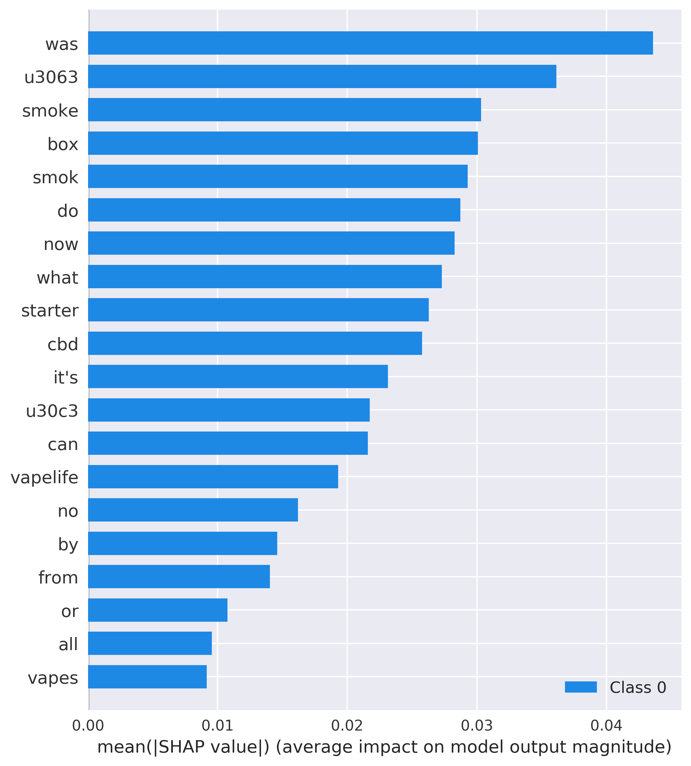


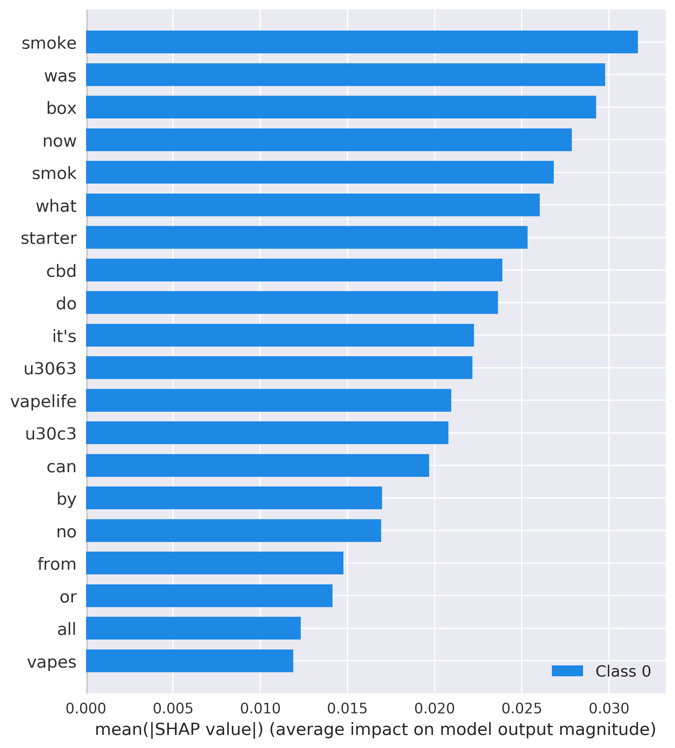

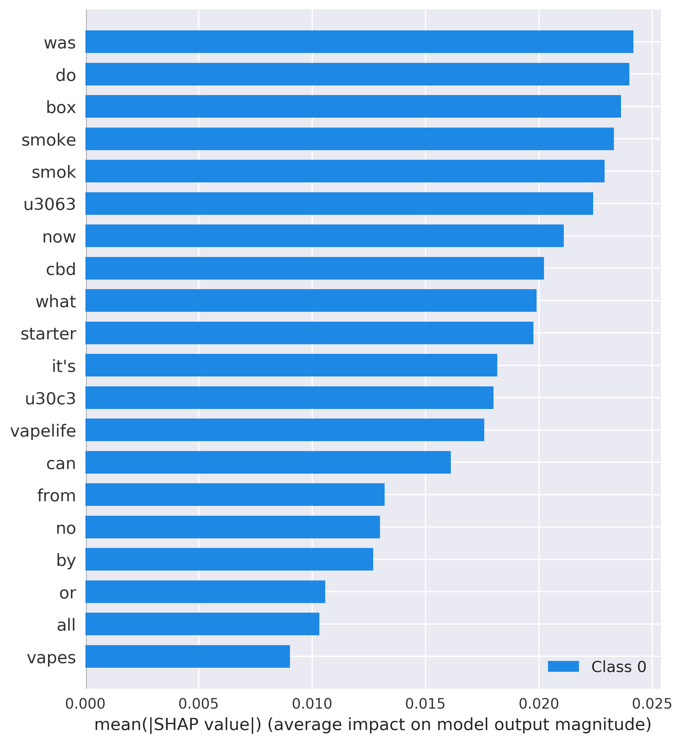


**Figure 2a.** The magnitude of 10 top-ranked features in traditional classifiers for commercial. Top row: LR and RF; bottom row: SVM and NB. (u3063, u30c3, u307e are Unicode characters)


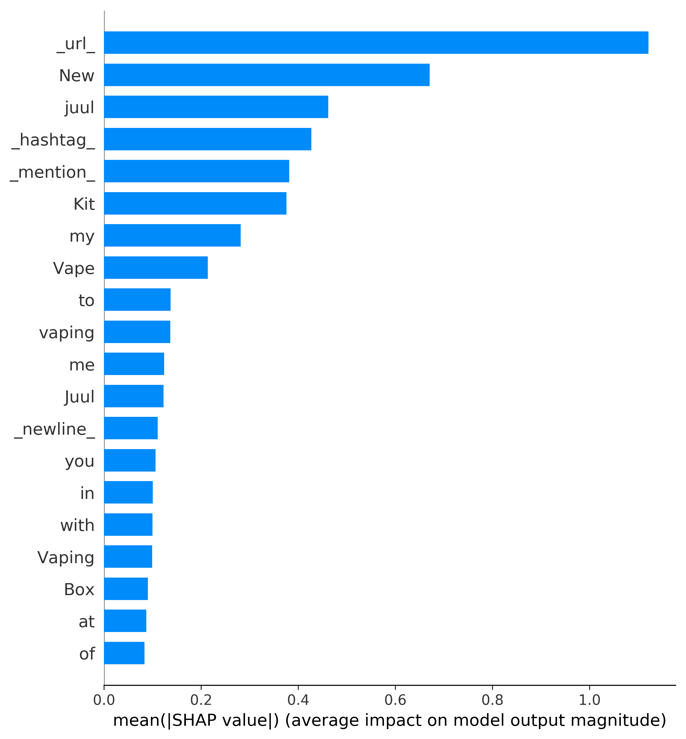

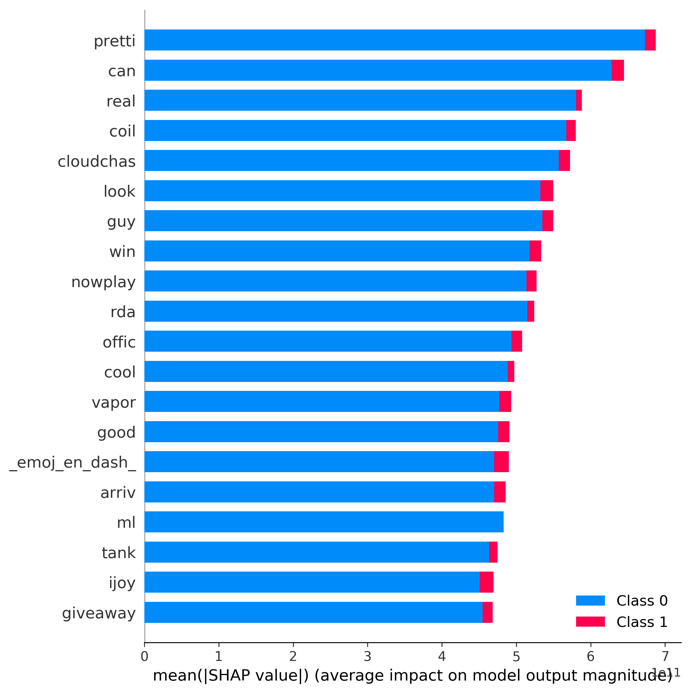


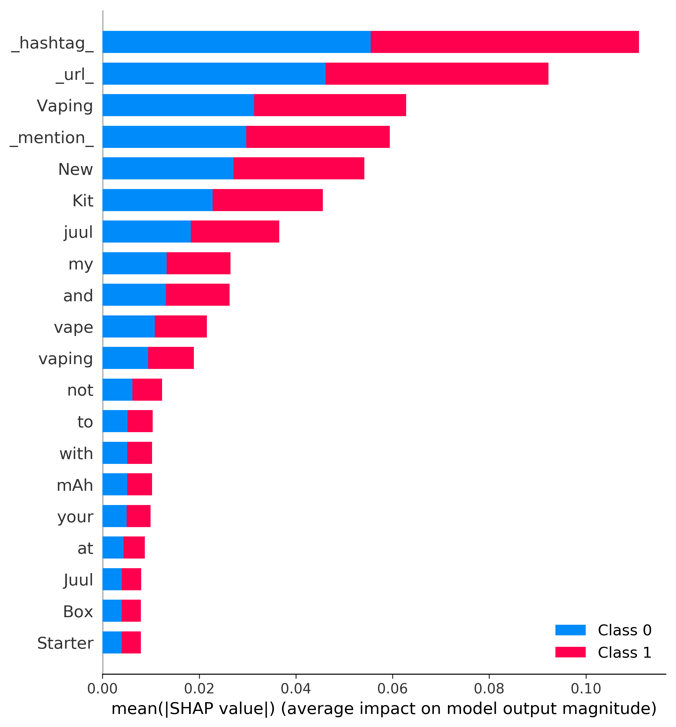

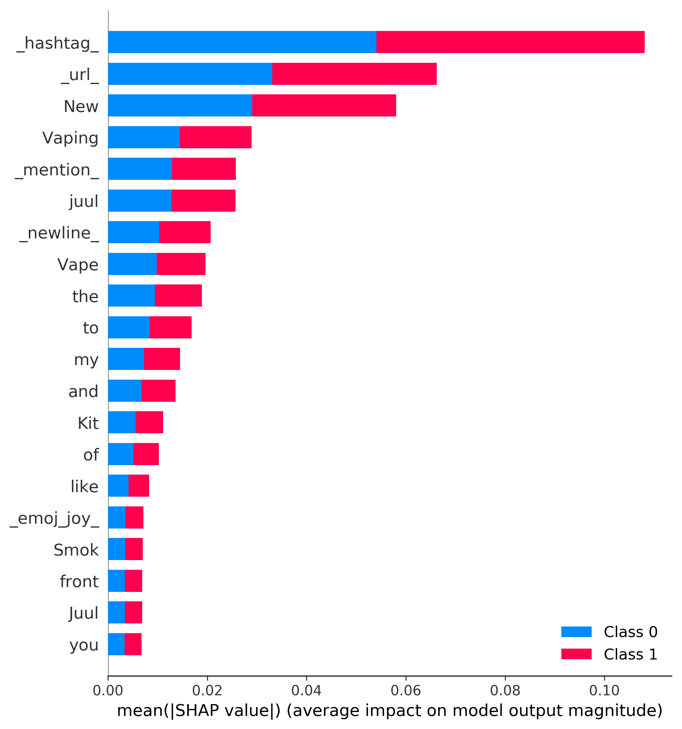


**Figure 2b.** The magnitude of 10 top-ranked features in deep learning classifiers with vaping-related word vectors for commercial. Top row: CNN and LSTM; bottom row: CNN-LSTM and BiLSTM.


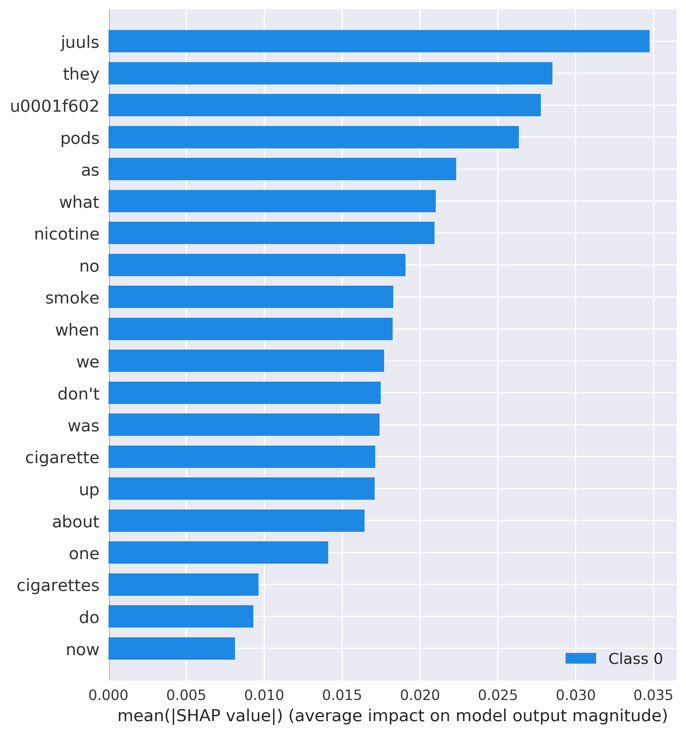

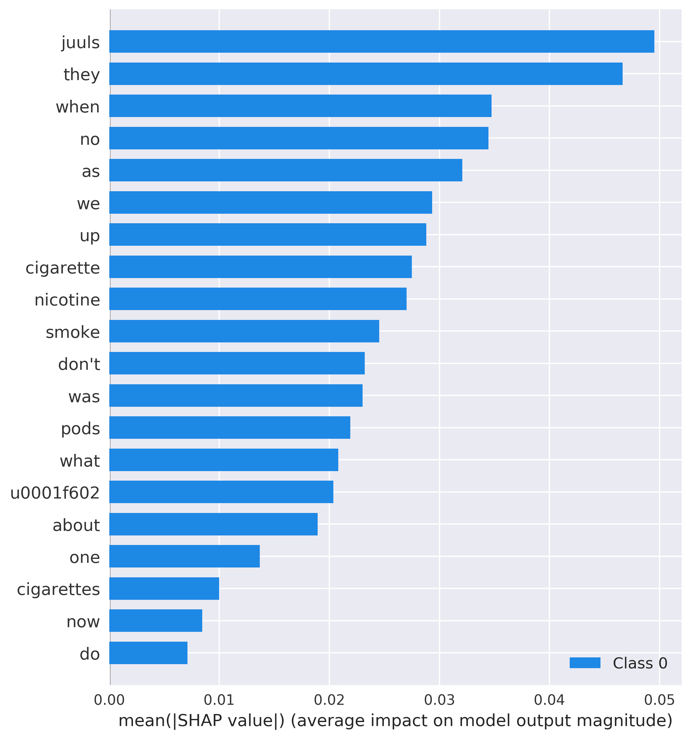


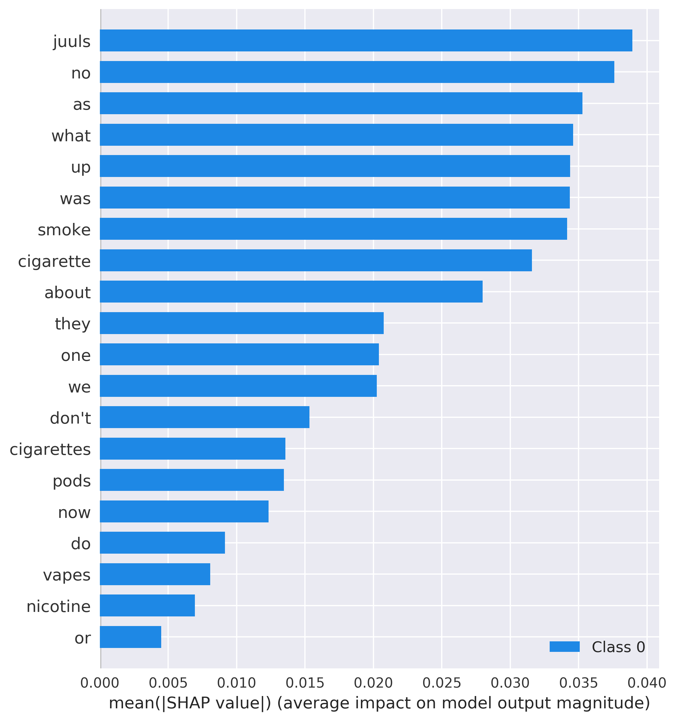

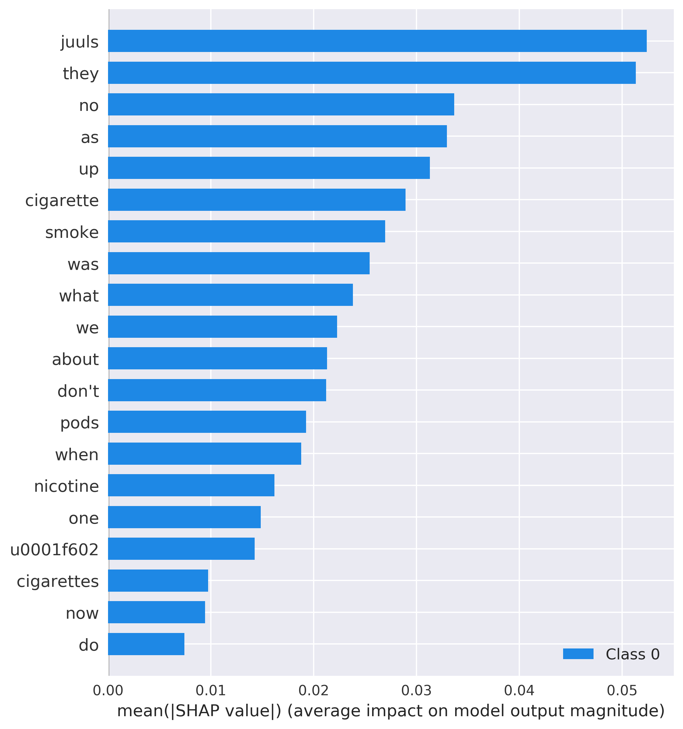


**Figure 2c.** The magnitude of 10 top-ranked features in deep learning classifiers with GloVe word vectors for commercial. Top row: CNN and LSTM; bottom row: CNN-LSTM and BiLSTM.


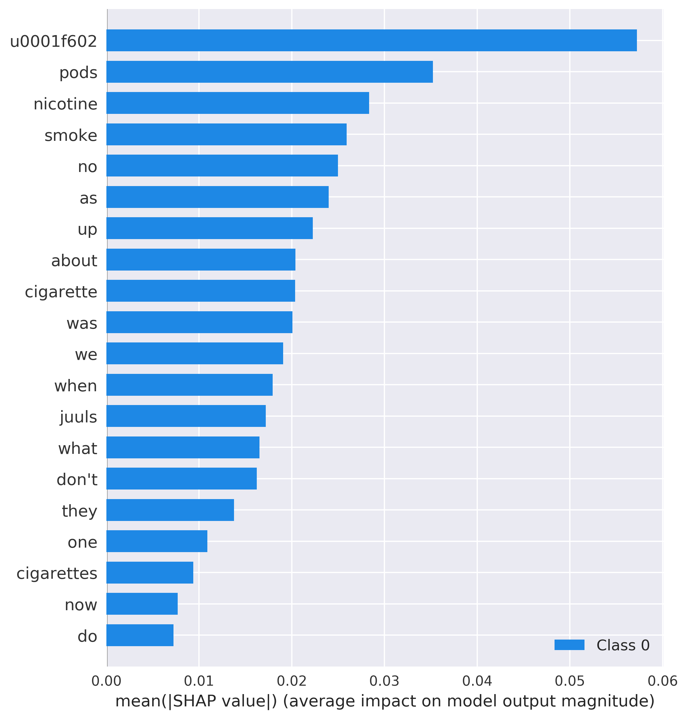

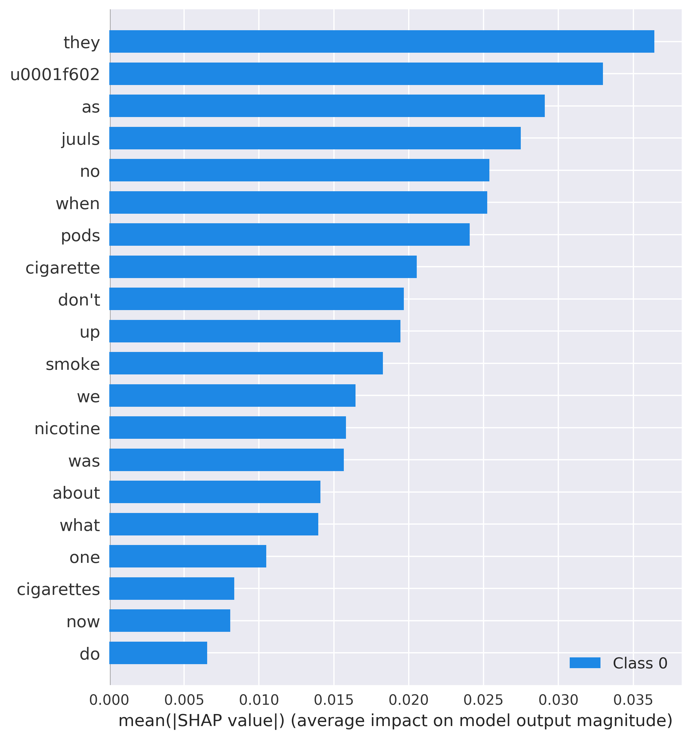


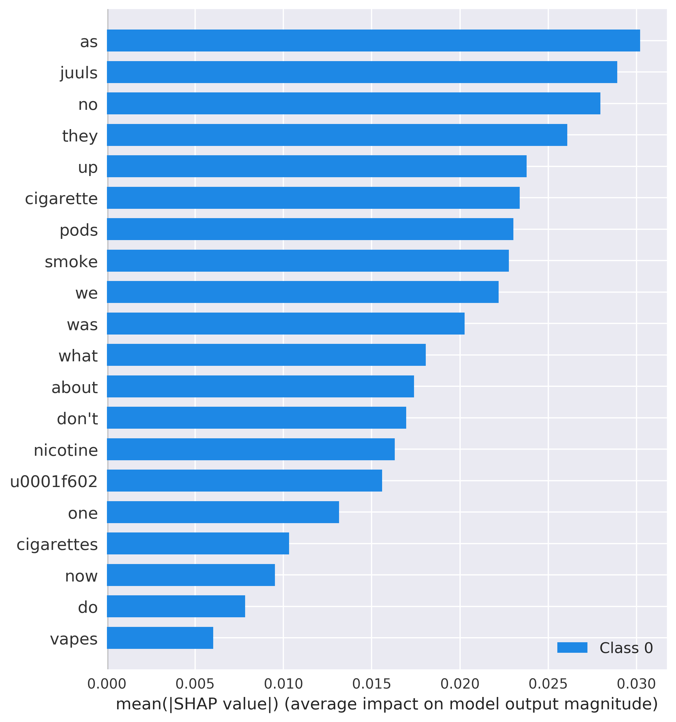

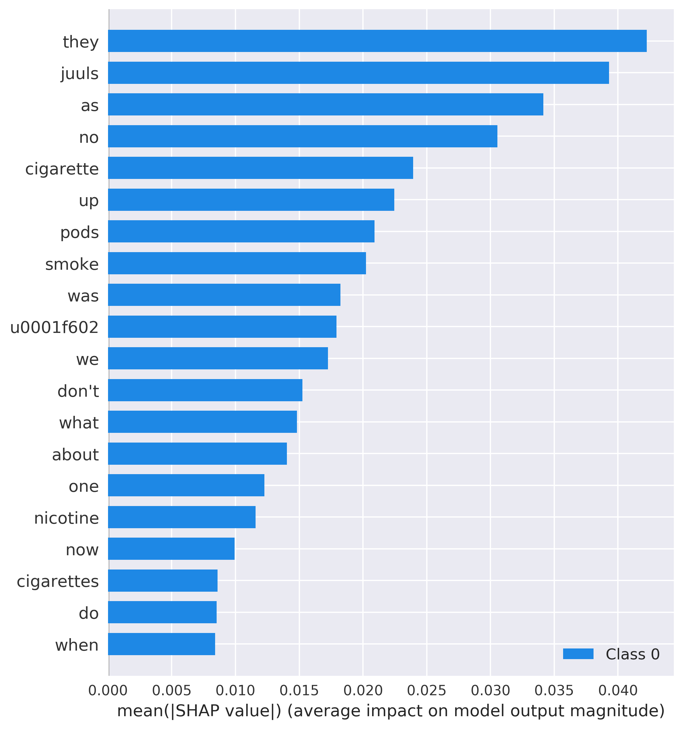


**Figure 3a.** The magnitude of 10 top-ranked features in traditional classifiers for sentiment. Top row: LR and RF; bottom row: SVM and NB.


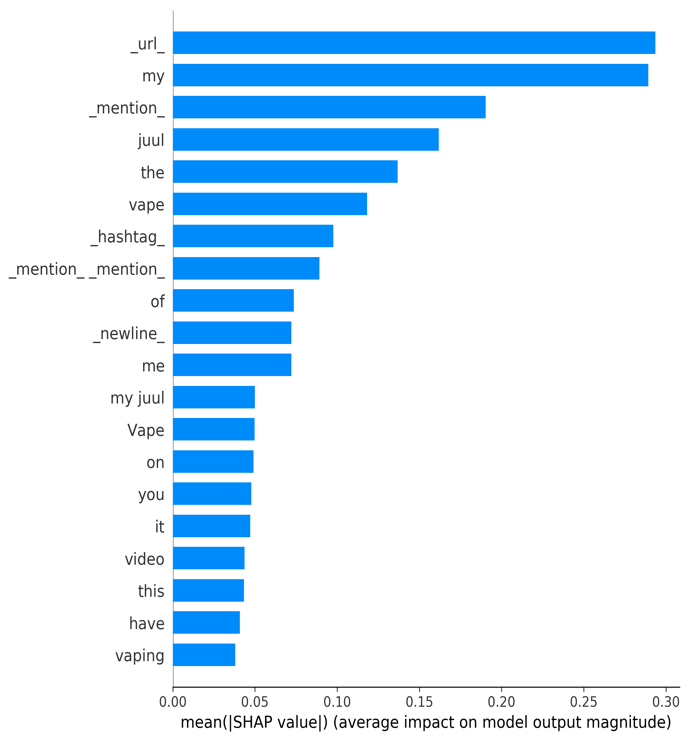

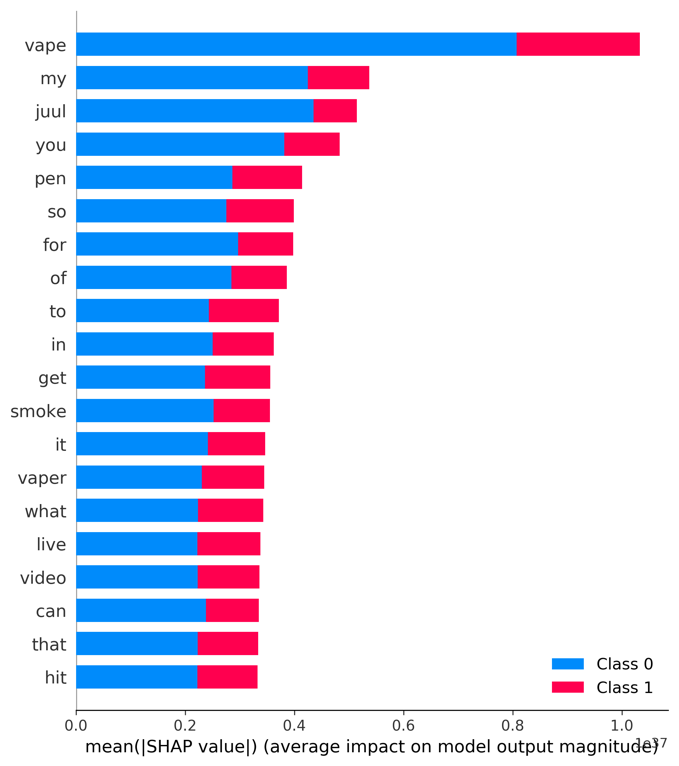


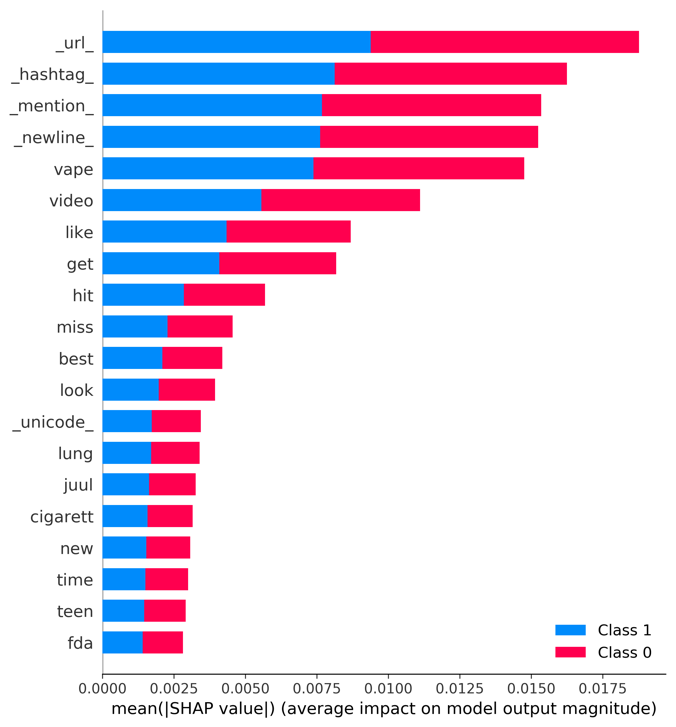

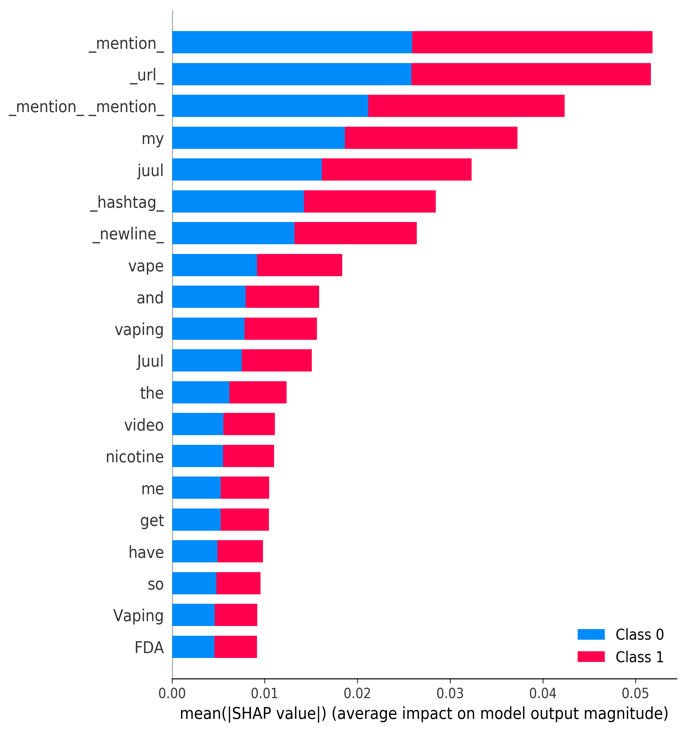


**Figure 3b.** The magnitude of 10 top-ranked features in deep learning classifiers with vaping-related word vectors for sentiment. Top row: CNN and LSTM; bottom row: CNN-LSTM and BiLSTM.


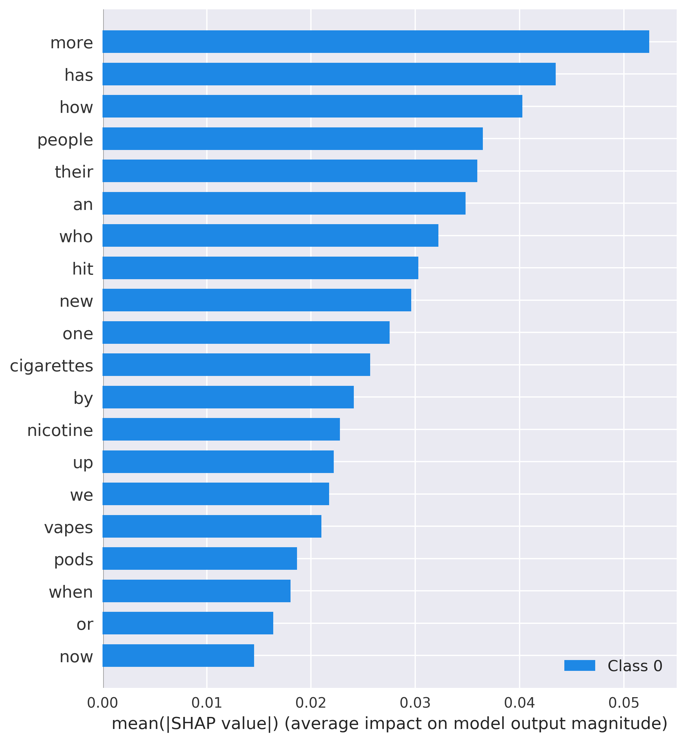

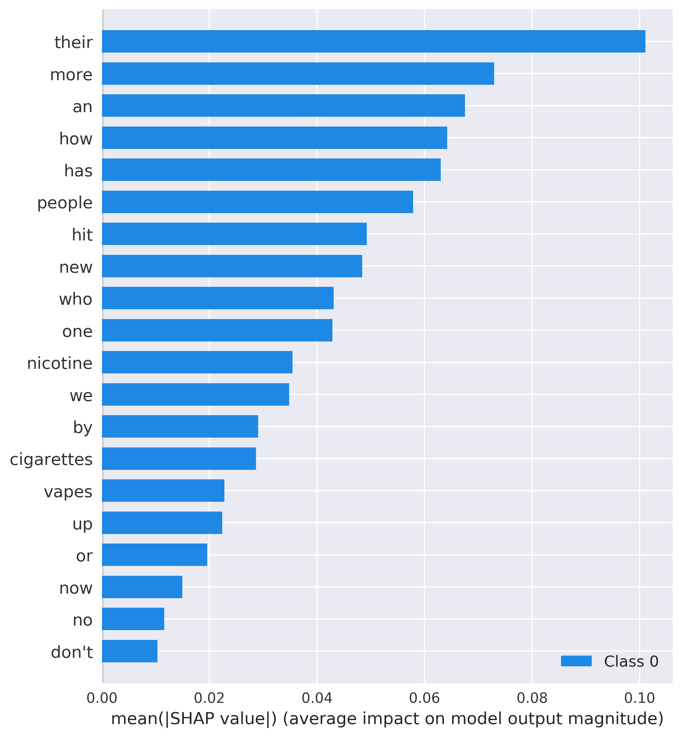


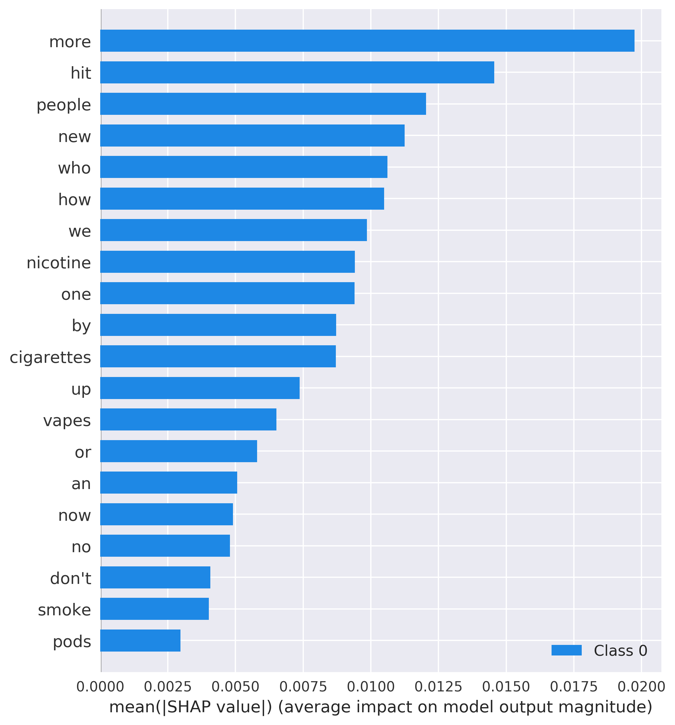

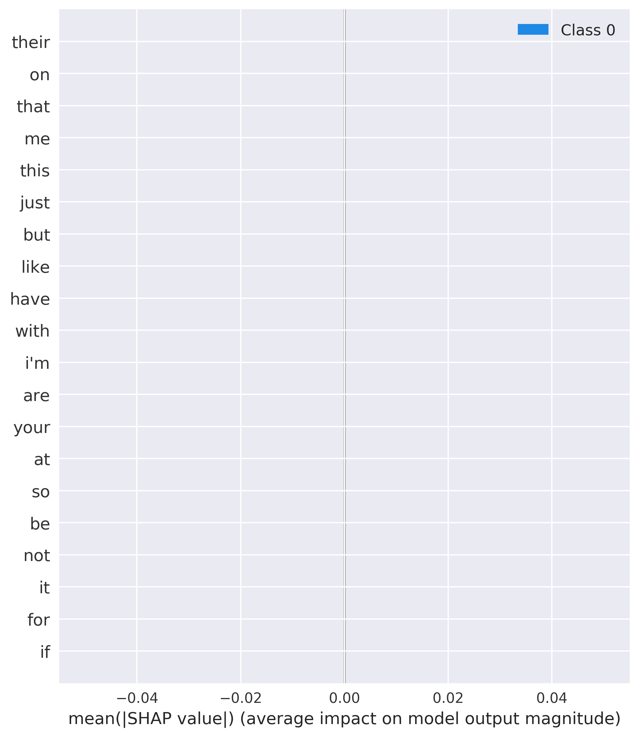


**Figure 3c.** The magnitude of 10 top-ranked features in deep learning classifiers with GloVe word vectors for sentiment. Top row: CNN and LSTM; bottom row: CNN-LSTM and BiLSTM.


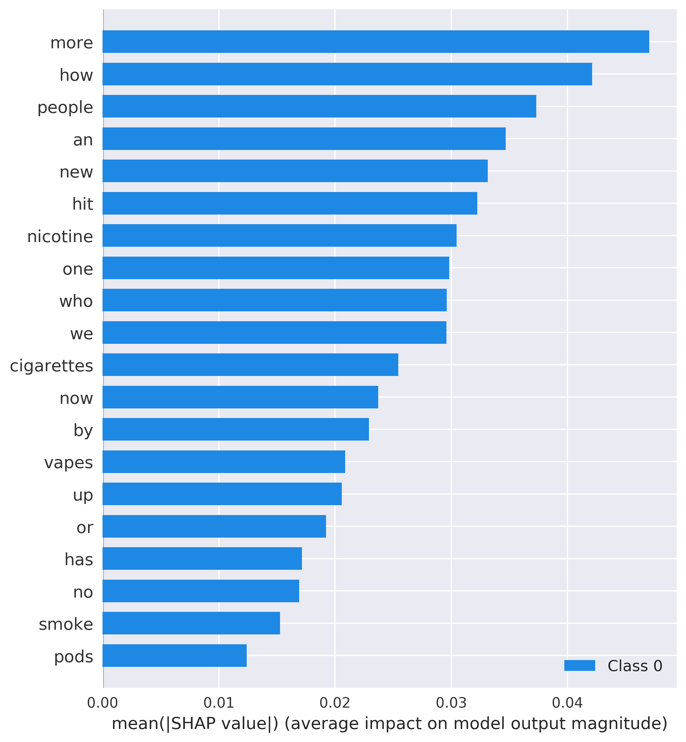

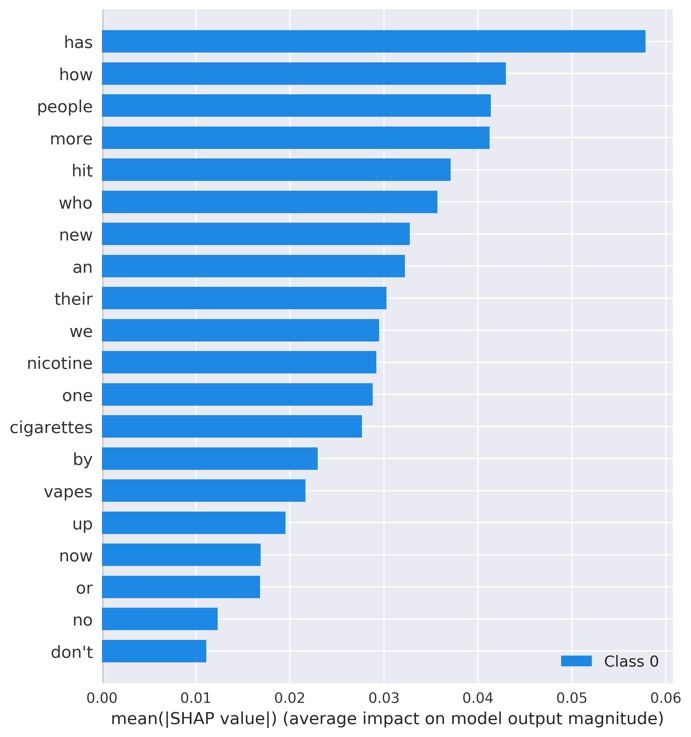


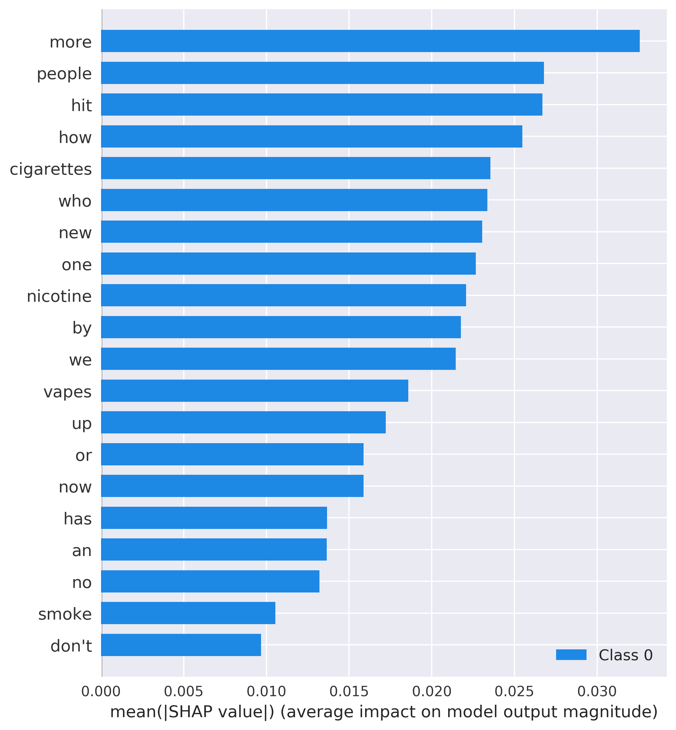

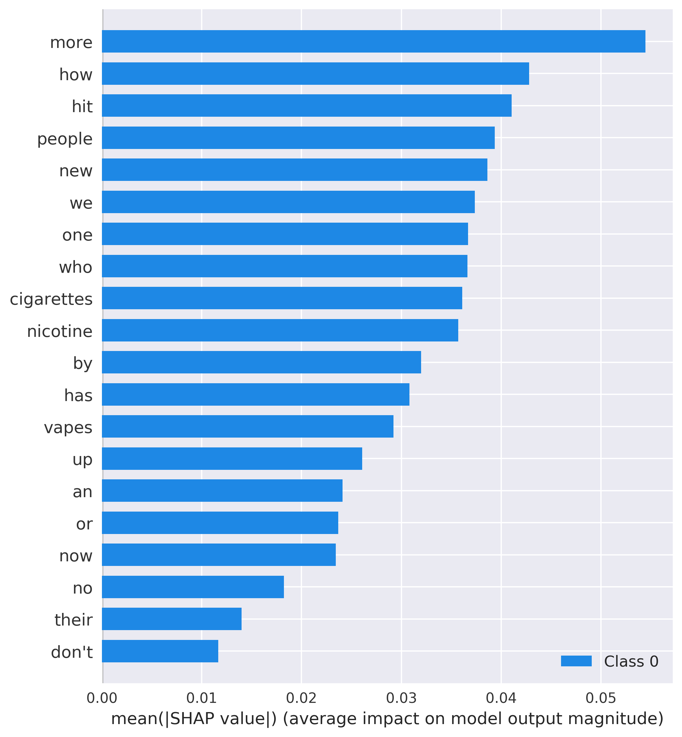

Supplement: Multimedia Appendix 1 [file jmir_v22i8e17478_app1.docx]
